# Supplementary material for: Cell Theranostics on Mesoporous Silicon Substrates
Source: Pharmaceutics. 2020 May 25;12(5):481. doi: 10.3390/pharmaceutics12050481 (PMC7284777; doi:10.3390/pharmaceutics12050481)
Supplement: Supplementary file 1 [file pharmaceutics-12-00481-s001.pdf]

pharmaceutics

Supporting Information

Maria Laura Coluccio [1,†] Valentina Onesto [1,†], Giovanni Marinaro [2,3], Mauro Dell'Apa [4], Stefania De Vitis [1], Alessandra Imbrogno [5], Luca Tirinato [1], Gerardo Perozziello [1], Enzo Di Fabrizio [6], Patrizio Candeloro [1], Natalia Malara [1,*] and Francesco Gentile [4,*]

1 Department of Experimental and Clinical Medicine, University Magna Graecia, 88100 Catanzaro, Italy; coluccio@unicz.it (M.L.C.); valentina.onesto@unicz.it (V.O.); stefania.devitis24@gmail.com (S.V.); tirinato@unicz.it (L.T.); gerardo.perozziello@unicz.it (G.P.); patrizio.candeloro@unicz.it (P.C.)
2 Institute of Process Engineering, Technische Universität Dresden, 01069 Dresden, Germany; giovanni.marinaro@kaust.edu.sa
3 Institute of Fluid Dynamics, Helmholtz-Zentrum Dresden-Rossendorf (HZDR), 01328 Dresden, Germany
4 Department of Electrical Engineering and Information Technology, University Federico II, 80125 Naples, Italy; mauro.dellapa@gmail.com
5 Emerging Materials & Devices, Tyndall National Institute, T12 R5CP, Cork, Ireland; alessandra.imbrogno@tyndall.ie
6 Department of Applied Science and Technology, Polytechnic University of Turin, 10129 Torino, Italy; enzo.difabrizio@polito.it
* Correspondence:Correspondence: nataliamalara@unicz.it (N.M.); francesco.gentile2@unina.it (F.G.)
† These authors contributed equally to this work.

Supporting Information 1. Determining the porosity of Mesoporous surfaces.

Acquired SEM images of MEP1 (Figure S1a) and MEP2 (Figure S1d) samples were analyzed with Matlab® to extract sample porosity (Table S1). Images were first converted to grayscale, preprocessed to enhance contrast by contrast-limited adaptive histogram equalization (CLAHE) and low-pass filtered to remove constant power additive noise before being binarized (Figures S1b,e) with Otsu's method. Morphological opening was performed to remove any small white noises in the image, and morphological closing to remove any small holes in the object (Figures S1c,f). The areas of all pores (black pixels) were summed and related to the total area of the considered image, according to Equation S1:

$$Porosity\ (\%) = \frac{\sum A_{pore}}{A_{tot}} 100 \quad (S1)$$

where A_{pore} is the value of the area of each pore, and $A_{tot}$is the total area of the acquired image.

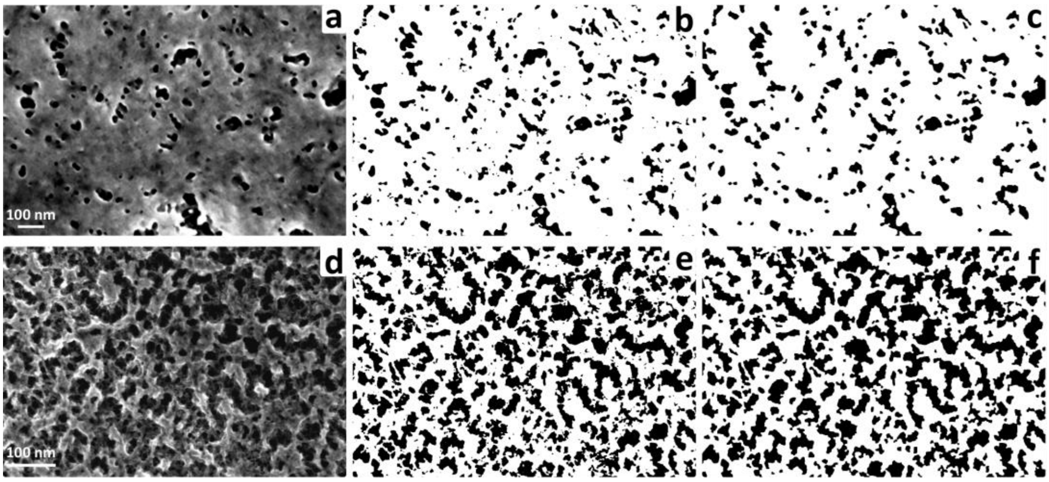

Figure S1. SEM micrographs of (**a**) MEP1 and (**b**) MEP2 substrates and corresponding binarization; (**c**–**f**) processed images after morphological opening and closing to remove noise and holes in the objects.

Table S1. Determined values of porosity for the mesoporous surfaces.

	Porosity	std
**MEP1**	12.47	1.91
**MEP2**	40.60	2.90

Supporting Information 2. Extracting Au NP diameter.

Au NP SEM micrographs (Figure S2a) were analyzed with Matlab® to extract Au NP diameter (Table S2). Images were first converted to grayscale, preprocessed to enhance contrast by contrast-limited adaptive histogram equalization (CLAHE) and low-pass filtered to remove constant power additive noise before being binarized with Otsu's method. Morphological opening was performed to remove any small white noises in the image, and morphological closing to remove any small holes in the object. The images were segmented by watershed transformation and a distance transform was used as segmentation function to split out the regions. Watershed transform is known for its tendency to oversegment an image because each local minimum, no matter how small, becomes a "catchment basin". For this reason we (i) filtered out tiny local minima using *imextendedmin* function (ii) modified the distance transform so that no minima occured at the filtered-out locations. This procedure is called "minima imposition" and was implemented via the function *imimposemin*.

With watershed segmentation, single nanoparticles were identified (Figure S2b). The diameter of the image regions were calculated through the *regionprops* function.

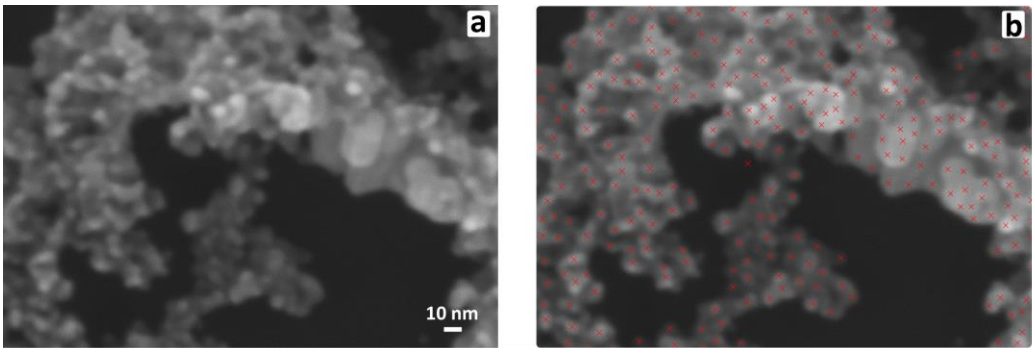

Figure 2. (**a**) SEM micrographs of Au NPs. (**b**) In red Au NP centroids, identified by watershed segmentation.

Table S2. Determined values of nanoparticle diameter.

Diameter	std
8.02	1.01
